# Voyages in Vacation.—X

**Published:** 1888-12-01

**Authors:** 


### Voyages in Vacation.—X.

By One in Search of Restful Restoratives.

### IMPRESSIONS OF ST. PETERSBURG.

*(Continued from page 119.)*

One of the first things to be done on landing at St. Petersburg was, of course, to secure some Russian money. Most of us had brought letters of credit, some had introductions in addition, and some had neither one nor the other. One gentleman had an introduction from a high personage to a firm of bankers, Messrs. Meyer. On presenting it, he asked for one or two small favours and civilities, which he ought to have received in the way of business without any letter of introduction at all. Not only did the partner make excuse in every case, but when his visitor became weary, and asked if they would cash him a circular note, he found that they not only charged him one-half per cent. commission, but gave him a rate of exchange which was about one-fifth of a rouble less than that of the day, so that their charges amounted to not less than 1¾ per cent. The Victorians who went to a strange bank or to an ordinary money-changer's obtained the full rate of exchange within a few kopecks, and without any deduction for commission at all. This action by the banker in question, who is responsible for the treatment we have described, was strongly animadverted upon by influential residents, who described him as a German whose methods were well known and appreciated in St. Petersburg. In any case we venture to believe it would have been impossible for any Russian to have been introduced, say by Messrs. Meyer, to any of their correspondents in London and to have received similar treatment. Any Englishman visiting Russia will do well to get letters of credit on the Discount Bank of St. Petersburg, which is centrally situated in the Nevsky Prospect, the manager and deputy-manager of which are gentlemen whose courtesy and knowledge of affairs make it a pleasure to meet them at any time.

There are excellent restaurants, and two comfortable hotels at least in St. Petersburg. The latter are the Hotel de l'Europe and the Hotel de France. At the time of our visit the Hotel de France was chiefly used by the members of the British Embassy, and its management and arrangements left little, if anything, to be desired. The reception and public rooms at the former hotel are considerably larger than those at the latter, but we prefer the Hotel de France on account of its compactness and comfort. The hall porter is an important personage in most European hotels, and especially in Russia, where the language is known to so few visitors. Those, therefore, who have no courier find themselves compelled to drive back to the hotel from each place they visit, because, as a rule, they have neither the experience nor the organising capacity to arrange a tour and to instruct the driver beforehand where they want to be taken to, and the order in which he is to visit the selected places. For this reason, the entrance hall of one of these hotels affords much amusement and interest to the cynic at all times, who is nothing if not hard on his fellow-man, and fellow-woman too for that matter.

The shops in St. Petersburg were disappointing: most of the articles were of foreign manufacture, and all the prices were excessively high. Owing possibly to the defective education of the people, signs are used by the tradesmen in the streets. Thus a butcher covers his house and shop-front with frescoes of joints, bullocks' heads, poleaxes, and other wares in which he deals. Loaves of bread, rolls, buns, cakes, tartlets, and so forth, illuminate the stucco in front of the baker's, whilst the greengrocer brings into play all the natural beauties of the flower garden with the sweet simplicity of the vegetables and fruits of the earth. This rough painting on the shop-fronts and sides of houses, and their many-coloured roofs, combined with the innumerable shrines which spring up almost everywhere throughout a Russian city, give it an air of attractiveness and a warmth of colour to which a resident in western countries is unaccustomed. Each shrine attracts its own little crowd of worshippers, and quite four out of every five persons who pass it make the sign of the cross, so that a devout driver, whilst threading his way through the city, will only have one hand free for the reins, because the other is continually occupied in making the sign of the cross as shrine after shrine is passed by in rapid succession.

One of the strangest sights is the funeral procession. Judging from the numbers which passed over St. Nicholas Bridge about half-past nine to half-past ten a.m., it must form a portion of the direct route to one of the chief cemeteries. There was sometimes an open hearse, on which lay the coffin covered by a pall, which was drawn by two horses at a rapid pace, and had no followers of any kind. Sometimes the hearse was preceded by mutes, priests, in full canonicals, and others, the first of whom often carry staves or lanterns, the bier and coffin being buried in flowers, although this was only in the case of some high personage, as we afterwards learnt, owing to the great difficulty of procuring flowers of any kind at St. Petersburg. The relatives and friends followed usually on foot, and the men always with bare heads. The most remarkable funeral, however, was that of a child. In this instance the mother and another woman were driven in a droschky, carrying the coffin, without covering of any kind, resting upon their knees. No doubt the friends in the last case were very poor, but the sight gave some of us rather a shock, and not without reason.

The canals, three in number, which run through the city, all cross the Nevsky Prospect, as the principal street is called, and are named the Moyka, the Fontooka, and the St. Catherine. They are chiefly used for purposes of trade, but a trip on one of the fast little steamers which dart about hither and thither throughout the day is remarkably interesting, owing to the fact that many of the finest houses are built upon their banks, and so several novel views are obtained, besides a fair insight into the life and habits of many of the citizens. The public buildings on the whole are disappointing when seen from the outside, although the palace of the Grand Duchess Helena and the famous Hermitage may be held to be more attractive than the others. The interiors of many of the palaces are a genuine surprise and pleasure to most visitors, but we must defer our description of them until next week.

It may be instructive and interesting by way of introduction to call attention in the present article to a novel feature attending the entrance and exit of every house or office in Russia. Within the entrance hall a servant is placed, whose duty it is to see that not only the hats and umbrellas, but the coats and wraps of each visitor are removed before entering the precincts of the house proper. It happened that we had to visit the Town Hall on more than one occasion in the same day, and in every instance hats, coats, etc., were removed and taken charge of. On receiving them back it was noticed that the courier invariably gave a fee to the attendant. On asking for an explanation, we were informed that no one would be allowed to enter a sitting room with his great coat on, and that if he did it would be taken as a personal insult, and would be resented in a manner more forcible than pleasant. It was further explained that it is recognised that the attendants shall receive, if they are not encouraged to take, tips from visitors, and we found this system prevailed to a much greater extent in Russia than even at home, where it is bad enough in many ways as many of us know too well. Its extent may be further gathered from the circumstance that there are upwards of two hundred rooms in the Winter Palace, and that those who were fortunate enough to secure an order of admission found the expense of seeing the whole was almost as great as that of visiting the Falls of Niagara in the old days. This arose from the fact that the rooms are divided into suites, each of which is placed in charge of attendants, and that it is the custom to give a certain fee to every attendant in every set of rooms which we visited. The character of the administration of the Imperial household, at any rate in former days, was indirectly shown by the circumstance that the present Tzar has given instructions for all the presentation salt dishes, many of which are of the most beautiful design and expensive character, to be suspended on the walls of the reception-rooms of the Winter Palace. Once placed there, they may be identified and recognised, as there was formerly good reason to believe that after a certain time very many of the most costly and handsome had a knack of mysteriously disappearing under the old system.

*(To be continued.)*

